# Simulation of BNNSs Dielectrophoretic Motion under a Nanosecond Pulsed Electric Field

**DOI:** 10.3390/nano11030682

**Published:** 2021-03-09

**Authors:** Yan Mi, Xin Ge, Jinyan Dai, Yong Chen, Yakui Zhu

**Affiliations:** State Key Laboratory of Power Transmission Equipment & System Security and New Technology, School of Electrical Engineering, Chongqing University, Chongqing 400044, China; 20181102038t@cqu.edu.cn (X.G.); 18846426220@163.com (J.D.); eecy@cqu.edu.cn (Y.C.); 202011021014@cqu.edu.cn (Y.Z.)

**Keywords:** nanosecond pulsed electric field, dielectrophoretic motion, local orientation, global arrangement, Maxwell stress tensor method

## Abstract

Using a nanosecond pulsed electric field to induce orientation and arrangement of insulating flake particles is a novel efficient strategy, but the specific mechanism remains unclear. In this study, the dielectrophoretic motion of boron nitride nanosheets (BNNSs) in ultrapure water under a nanosecond pulsed electric field is simulated for the first time. First, the simulation theory is proposed. When the relaxation polarization time of the dielectric is much shorter than the pulse voltage width, the pulse voltage high level can be considered a short-term DC voltage. On this basis, the Arbitrary Lagrangian–Euler (ALE) method is used in the model, considering the mutual ultrapure water–BNNS particles-nanosecond pulsed electric field dielectrophoretic interaction, to study the influence of different BNNSs self-angle α and relative angle β on local orientation and global arrangement. The particles are moved by the dielectrophoretic force during the pulse voltage high level and move with the ultrapure water flow at the zero level, without their movement direction changing during this period, so the orientation angle and distance changes show step-like and wave-like curves, respectively. The model explains the basic mechanism of dielectrophoretic motion of BNNSs under a pulsed electric field and summarizes the motion law of BNNSs, providing a reference for subsequent research.

## 1. Introduction

Applying an electric field to align small particles is considered to be the most direct and simplest control method [1]. Among them, the form of electric field is divided into DC electric field, AC electric field and pulse electric field [2,3]. Our group has previously used microsecond pulsed electric fields to study the orientation of boron nitride nanosheets (BNNSs) in epoxy resin. The results showed that a 20 kV pulse voltage can make the orientation degree of BNNSs reach 86%, indicating that almost all BNNSs have been oriented along the electric field direction [4]. Cho et al. used a 40 kV nanosecond pulse voltage to successfully induce the alignment of BNNSs [5,6]. Therefore, the use of a high-intensity pulsed electric field to induce the orientation and arrangement of insulating flake particles can overcome the breakdown field strength of the matrix and greatly save energy [7], which is a novel and efficient strategy. However, the induction mechanisms and laws are not yet clear. Simulation can be used to explore its mechanisms and laws, to further the understanding of the process.

Under a nanosecond pulsed electric field, the particles are subjected to a dielectrophoretic force, so the particles undergo a dielectrophoretic motion of local orientation and global arrangement, and finally form chains [8]. Pohl [9] first used the equivalent dipole moment method (EDM) to develop the existing dielectric force calculation, but this method is not accurate when the gap between particles is equal to or smaller than the particle size [10]. In addition, the Clausius–Mossotti (CM) factor in the EDM only applies to spherical particles. For particles with different aspect ratios, Ai and Qian [11,12,13,14] used the Arbitrary Lagrangian–Eulerian (ALE) methods to simulate the two-dimensional liquid–solid dielectric interaction of a pair of particles under a uniform DC electric field. At the same time, they used microfluidic devices to demonstrate that the predicted particle trajectory shifts due to dielectrophoretic force show quantitative agreements with the existing experimental data. ALE is based on the Maxwell stress tensor (MST) method [15], which considers the fluid–particle–electric field interaction and accurately simulates the dielectrophoretic motion of particles in the fluid system. The MST method is used to solve the dielectrophoretic force on particles and is considered to be the most rigorous method in the calculation of the dielectrophoretic force [16,17,18]. In addition, Ai and Qian [19] also used this method to simulate the dielectric interaction between particles in an AC electric field. However, no research using this method to simulate and analyze the force on particles under a nanosecond pulsed electric field has been presented. Therefore, simulating the dielectrophoretic motion of nonspherical particles under a nanosecond pulsed electric field is of great significance for solving practical problems.

Due to the narrow pulse width of the nanosecond pulse voltage, in this study, the pulse width is 500 ns, the polarization time of the dielectric is first compared with the pulse width of the pulse voltage field, and a theory for simulating the dielectrophoretic motion of particles under a nanosecond pulsed electric field is proposed. Considering that the distribution of BNNSs in the fluid is highly disordered, their own initial tilt angle and the relative angles of multiple BNNSs are disordered, and finding the law of motion in a simulation is difficult. Therefore, taking two BNNS particles as an example, based on the finite element method (FEM) and ALE method and fully considering the BNNS particles-ultrapure water fluid-nanosecond pulsed electric field interaction, the MST method is then used to solve the dielectrophoretic force on BNNSs and simulate the influence of different positions on the local orientation and global arrangement of BNNSs under a nanosecond pulsed electric field, providing guidance for subsequent research.

## 2. Simulation Methods

### 2.1. Theoretical Study of Dielectrophoretic Motion under a Nanosecond Pulsed Electric Field

**Figure 1:**
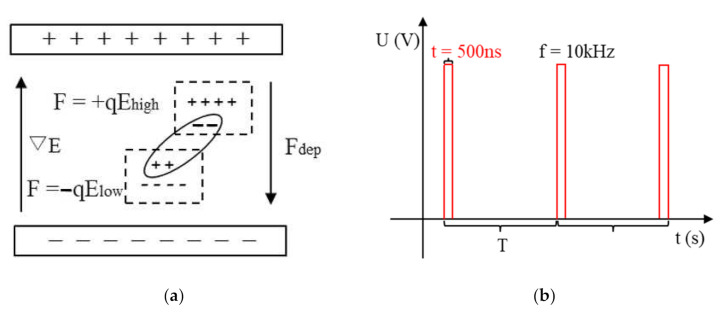
(**a**) Schematic diagram of particle polarization; (**b**) pulse voltage waveform.

The fluid in this simulation is ultrapure water with a permittivity ε_fl_ = 81ε_0_, where the subscript fl represents fluid and ε_0_ is the permittivity in vacuum. Elliptical particles with different aspect ratios are abstracted two-dimensional patterns of insulated BNNSs, with a permittivity ε_p_ = 4ε_0_, where the subscript p represents the BNNS particles. When electrically neutral insulating particles are suspended in a dielectric solution and an electric field is applied, both the particles and the dielectric solution will be polarized and form bound charges on their surface. According to the difference in the permittivity of the particle and the dielectric solution, the amounts of bound charge inside and outside the particle interface will be different, and dielectrophoretic motion will occur under the electric field. At this time, ε_fl_ > ε_p_ indicates that the polarizing ability of the ultrapure aqueous solution is stronger, and more bound charges gather on one side of the solution, as shown in Figure 1a. The nonuniform distribution of charge leads to a difference in the charge density between the two ends of the particles. At this time, under the nanosecond pulsed electric field, the BNNS particles undergo negative dielectrophoretic motion and move in the direction of low field strength. In the simulation, the pulse voltage pulse width is t = 500 ns and the frequency is f = 10 kHz, as shown in Figure 1b.

However, the formation of dipoles by dielectric polarization belong to slow relaxation polarization, that is, the Debye relaxation process. The Debye relaxation process has the characteristic of an e-exponential change with time, and the essence is that all charges move with the average time of *τ*. Therefore, when a nanosecond pulse voltage is applied, its relaxation polarization time must be compared with the nanosecond pulse width to determine whether the relaxation polarization process can be completed during the high level of the nanosecond pulse and reach the stable relaxation pole strength.

According to the literature [20], at low frequencies, the ultrapure water dielectric conforms to the single Debye relaxation model. Therefore, the real and imaginary parts of the relative permittivity can be fitted using a single relaxation Cole–Cole model. In the Cole–Cole model, the absorption band frequency ϖ = 18 GHz of the highest point of the semicircle can be obtained, so the relaxation polarization time *τ* of ultrapure water is

(1) $$\tau \;=\;\frac{1}{\varpi }\;=\;\frac{1}{2\pi f}\;=\;\frac{1}{2\times 3.14\times 18\times 10^{9}}\approx 0.0088\;\mathrm{ns}$$

From the above calculation, the relaxation polarization time of ultrapure water of 0.0088 ns is far less than the nanosecond pulse width of 500 ns. Therefore, the effect of the relaxation polarization time of the water molecules on the dielectrophoretic motion of particles can be ignored in the simulation. Hexagonal BNNSs are composed of boron and nitrogen atoms distributed in a dense sp2 hexagonal lattice [21]. The material exhibits significant ionic characteristics, which causes the electrons in the π bond to not delocalize [22], so almost no relaxation phenomenon occurs. At 10 GHz, the permittivity is still stable [23]. Therefore, the instantaneous polarization time is also far less than the nanosecond pulse width of 500 ns.

The above analysis shows that during the high level of the pulsed electric field, BNNSs and ultrapure water have completed the relaxation polarization process and reached a stable state, so the high level period of the pulsed electric field can be regarded as a short-term DC electric field.

### 2.2. Mathematical Model

This section describes the mathematical model of the orientation and arrangement of insulated elliptical particle BNNSs under a nanosecond pulsed electric field. The ALE method is used to solve the flow field, electric field and particle motion simultaneously and obtain the dielectrophoretic force by directly integrating the MST on the BNNSs surface without any assumptions.

**Figure 2:**
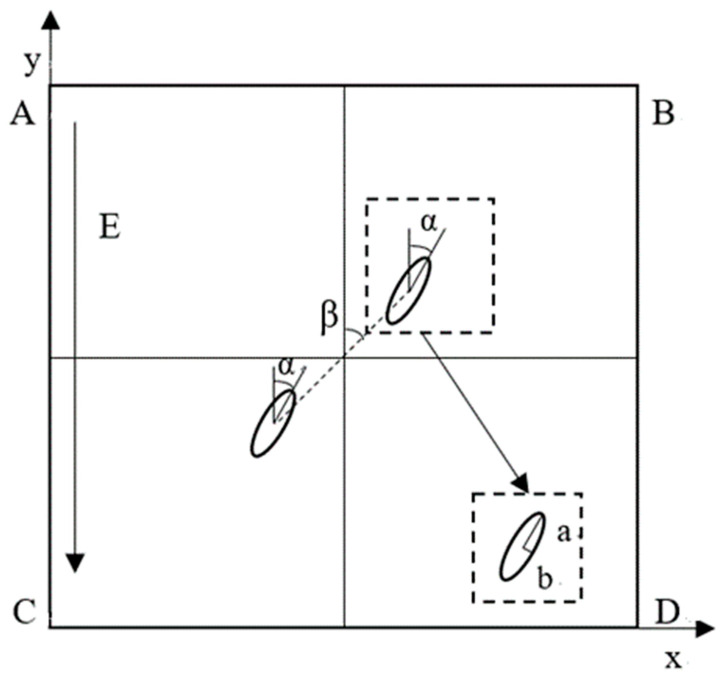
Simulation geometry model.

The simulation geometry model is shown in Figure 2. The square is an incompressible ultrapure water dielectric solution containing a pair of insulated BNNSs, with fluid density ρ = 1000 kg/m^3^, dynamic viscosity η = 0.001 Pa·s, and permittivity ε_fl_ = 81ε_0_. According to the actual size of the BNNSs in the previous experiment, the long axis radius and short axis radius of the elliptical particles in the simulation are set to α = 5 µm and b = 1.6 µm, respectively, and the permittivity is ε_p_ = 4ε_0_. The relative angle of the two BNNSs is β, the self-angle is α, the relative center distance is d, and the nanosecond pulsed electric field E is applied along the *y*-axis. The side length of the square ABCD is 20a = 100 µm, which is large enough to ensure that the outer boundary of the solution domain will not be significantly affected by the particles.

When a nanosecond pulsed electric field is applied, the MST of the BNNSs is [17]:

(2) $$T_{E}=\varepsilon_{f}\left[E_{f}E_{f}-\frac{1}{2}(E_{f}\cdot E_{f})I\right],$$

where $E_{fl}$ is the nanosecond pulsed electric field intensity received by the particles, $\varepsilon_{fl}$ is the permittivity of the ultrapure water solution, and I is the unit tensor.

The boundary integration of the MST outside the BNNSs provides the dielectrophoretic force $F_{dep}$ and torque $L_{dep}$ of the particles as:

(3) $$F_{dep}=\int_{\partial \Omega }(T_{E}\cdot n)ds,$$

(4) $$L_{dep}=\int_{\partial \Omega }(T_{E}\times (x_{si}-x_{pi})\cdot n)ds,$$

where $T_{E}$ is the MST and $x_{si}$ and $x_{pi}$ are the position vectors of the surface and center of the i-th particle, respectively.

The fluid is in a static state at the initial moment. When the BNNSs start to move under the action of the dielectrophoretic force, this will cause flow of the ultrapure water. The continuity equation and governing equation of fluid are expressed as:

(5) ∇u = 0,

(6) $$\rho_{f}[\frac{\partial u}{\partial t}+(u\cdot \nabla )u]-\nabla \cdot \eta_{f}(\nabla u+{\left(\nabla u\right)}^{T})+\nabla p\;=\;0,$$

where $\rho_{f}$ is the fluid density, $\eta_{f}$ is the dynamic viscosity of the fluid, and p is the fluid pressure. An open boundary condition is set on the boundary ABCD so that the fluid can enter and leave the calculation domain through the boundary. Assuming that the density of the particles is consistent with the density of the fluid, the effects of gravity and buoyancy do not need to be considered. The motion of BNNSs is mainly affected by the dielectrophoretic force and fluid resistance, producing local orientation and global arrangement.

In the model, the dynamic stress tensor $T_{H}$ of the flow field is:

(7) $$T_{H}=\eta_{f}(\nabla u_{i}+{\left(\nabla u_{i}\right)}^{T}),$$

(8) $$u_{i}={\;U}_{pi}+\omega_{pi}\times (x_{si}-x_{pi}),$$

where $u_{i}$ is the flow velocity on the surface of particle *i*. $U_{pi}$ and $\omega_{pi}$ are the translation speed and rotation speed of the *i*-th particle, and $x_{si}$ and $x_{pi}$ are the position vector of the surface and center of the *i*-th particle, respectively.

Therefore, the Stokes fluid resistance $F_{H}$ and torque $L_{H}$ received by the particles are:

(9) $$F_{H}=\int_{\partial \Omega }(T_{H}\cdot n)ds,$$

(10) $$L_{H}=\int_{\partial \Omega }(T_{H}\times (x_{si}-x_{pi})\cdot n)ds.$$

In summary, the total force exerted on the *i*-th particle includes the Stokes fluid force and the dielectrophoretic force, which are obtained by integrating the fluid dynamic force stress tensor ($T_{H}$) and the MST ($T_{E}$) on the surface of the *i*-th particle, respectively. According to Newton’s second law and the Euler equation for describing the translation and rotation of particles:

(11) $$m_{pi}\frac{dU_{pi}}{dt}=F_{dep}+F_{H},$$

(12) $$I_{pi}\frac{d\omega_{pi}}{dt}=L_{dep}+L_{H},$$

where $m_{pi}$ is the mass of particle *i* and $I_{pi}$ is the moment of inertia of particle *i*, so the trajectory of the *i*-th particle is calculated by the following formula (ALE):

(13) $$X_{pi}(t)=X_{pi}(0)+\int_{0}^{t}U_{pi}(t)dt^{\prime },$$

(14) $$\alpha_{pi}(t)=\alpha_{pi}(0)+\int_{0}^{t}\omega_{pi}(t^{\prime })dt^{\prime },$$

where $X_{pi}(t){\;=\;(x}_{pi},y_{pi})$ is the center position of the *i*-th particle and $\alpha_{pi}(t)$ is the orientation angle of the *i*-th particle.

The finite element software COMSOL Multiphysics is used to implement and solve mathematical models. The two-way coupling of the particle-fluid-electric field model is solved by the ALE method using a time-dependent solver. The modules include AC/DC, fluid-structure coupling and a mobile grid, and the Maxwell stress tensor (MST) formula is added to the AC/DC module. The particles start to move under the dielectrophoretic force. A mobile grid is used to track the fluid domain where the grid deforms freely. The grid of the particle domain is fixed, and the deformation of the fluid domain depends on the trajectory and direction of the particle movement, which is the Equations (13) and (14). At this time, the deformation area of the fluid domain is automatically remeshed for partitioning to avoid the inaccurate solution caused by deformation of the mesh. Regarding the relevant details of the model, to accurately use the MST method to calculate the dielectrophoretic force and torque of the pulsed electric field acting on the BNNSs, a fine grid is applied on the surface of the particles and around the larger curvature, and set boundary layer conditions at the particle boundary. The total number of grid cells in the entire domain is about 27,000, and the number of cells on the surface of each particle is about 560 to obtain stable simulation results.

## 3. Simulation Results and Discussion

### 3.1. Distribution of the Electric Field Intensity

The uneven distribution of the field strength causes the particles to be subjected to a dielectrophoretic force, resulting in local orientation and global arrangement movement. According to the previous analysis, the BNNSs undergo negative dielectrophoretic motion in ultrapure water, and the particles move in the direction of low field strength [24].

**Figure 3:**
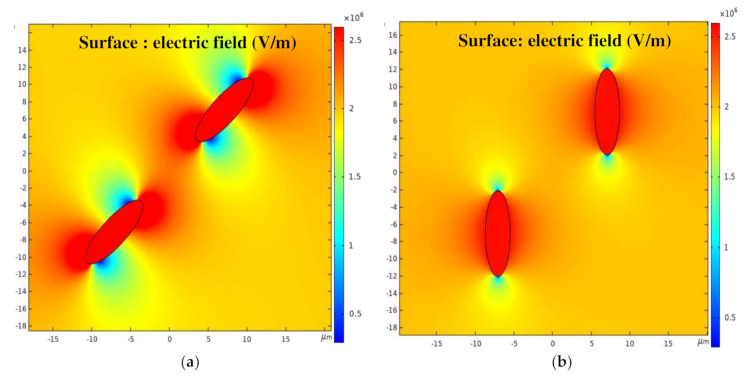
(**a**) Electric field intensity distribution cloud map (local orientation); (**b**) electric field intensity distribution cloud map (global arrangement).

Figure 3 shows a cloud map of the electric field intensity distribution for BNNSs in an ultrapure water fluid. The upper left and lower right ends of the negative dielectrophoresis BNNSs appear as low electric field intensity regions during orientation, so the particles moving in the low electric field direction appear to be oriented along the electric field direction. After the orientation is completed, the upper and lower ends of the particles appear as low electric field strength areas, and the left and right ends appear as high electric field strength areas. The external uniform electric field is the same everywhere in the absence of particles, and the interior of the particles is a high electric field intensity area, so the two particles begin to move towards each other in negative dielectrophoresis, as shown in Figure 3b.

The uneven field strength distribution is the root cause of particle dielectrophoretic motion. The influence of different self-angles and relative angles are discussed below, and the motion law of BNNSs at different positions under a nanosecond pulsed electric field is obtained.

### 3.2. Influence of Self-Angle α on Local Orientation

The dielectrophoretic motion of two BNNSs under an electric field involves the process of local orientation and global arrangement. According to the literature [25], global arrangement of particles takes more time than local orientation; the particles are first aligned with the electric field, and then, they are arranged in chains. Larger initial angles and larger initial relative angles and center distances often require more time to form a stable chain. For example, particles with α = 45° first undergo counterclockwise local orientation from 45° to 90° and then spend most of their time globally arranging into chains. Therefore, when studying the global arrangement of particles, assuming that the particles have completed their local orientation is reasonable. When studying the local orientation process of particles, assuming that the particles do not have a global arrangement is reasonable. Therefore, we separate the two processes and then separately study the local orientation process and the global arrangement process of the two BNNS particles to obtain clearer rules.

**Figure 4:**
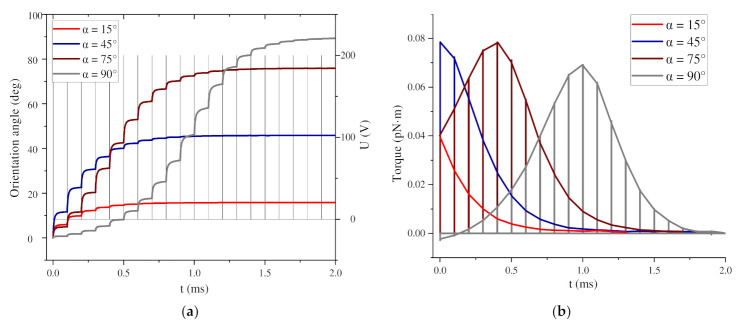
(**a**) Change in the boron nitride nanosheets (BNNSs) orientation angle with nanosecond pulse voltages; (**b**) change in the BNNS torque with nanosecond pulse voltages.

In this section, the local orientation process is first studied, and the motion law of two BNNS particles at different initial angles α is simulated. Set α = 15°, 45°, 75°, and 90°. When α = 0°, the long axis of the particle is parallel to the electric field direction, and no local orientation process occurs at this time. Set d = 2.0d* and β = 45°, both of which are fixed values, where d* is the diameter of the long axis of the particle, with d* = 10 µm. Figure 4 shows the changes in the torque and orientation angle of a pair of BNNS particles under a nanosecond pulsed electric field when the particles have different α.

Figure 4 shows the curves of the orientation angle and torque changes under the first 20 pulse voltages. After the pulsed electric field is applied for 2 ms, the BNNSs with different α have basically completed the orientation. As α increases, the time required for orientation gradually increases. Among them, the BNNSs with α = 90° have the longest orientation time because the angle between their initial angle and the electric field direction is the largest. Under each pulse voltage, the change in the orientation angle under different α conditions is also different, which depends on the torque change of the particles when the electric field is applied. When α = 15° and α = 45°, the torque on the particles decreases with the action of the pulse voltage, so the change in the orientation angle also gradually decreases. When α = 75° and α = 90°, the torque on the particles first increases and then decreases, so the change in orientation angle also first increases and then decreases. The change in the orientation angle of the BNNSs is determined by the torque received by the particles, and the torque changes symmetrically around 45°. The torque is the product of the dielectrophoretic force and the arm of the force, so in the orientation process, the change in torque includes the change in the dielectrophoretic force.

In particular, since the initial positions of α = 15° and α = 75° are symmetrical about 45°, the initial torque is the same, but once the BNNS rotates, the torque will immediately become different. α = 45° is initially at the center of symmetry. At this time, the torque reaches the maximum, so the initial orientation angle change is the largest, which can reach approximately 11.62°. When α = 90°, because this corresponds to a special position perpendicular to the electric field direction, the BNNS will swing about the origin under the action of the ultrapure water fluid, and the initial torque is small, so the initial orientation angle change is also very small.

**Figure 5:**
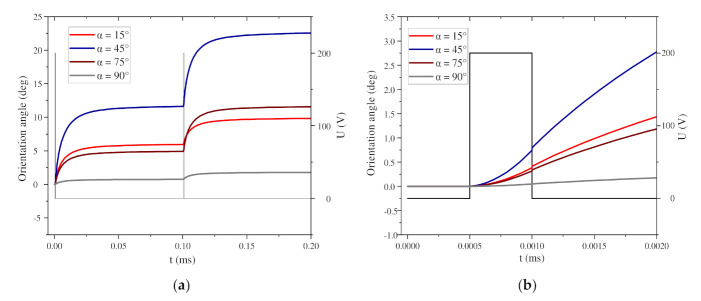
(**a**) Change in orientation angle under 2 nanosecond pulse voltages; (**b**) change in orientation angle during 1 nanosecond pulse voltage.

**Figure 6:**
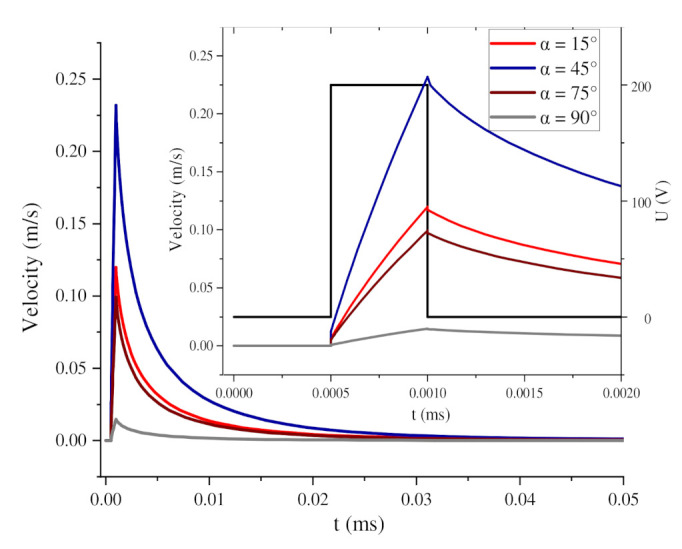
Change in movement velocity during 1 pulse voltage.

Figure 5a shows the change in the orientation angle of the BNNSs under two pulse voltages, and Figure 5b shows the change in the orientation angle during the first pulse voltage. The pulse voltage high level period is regarded as a short DC electric field. At this time, the orientation angle of the particles begins to increase. When the high level ends, the orientation angle continues to increase, but the growth rate decreases, and Figure 5a shows the stepped waveform. The reason for this phenomenon is shown in Figure 6. When the BNNSs are oriented, they will move under the nanosecond pulsed electric field and flow field. During the high level period, the velocity of the particles increases sharply and drives the flow of the surrounding fluid. During the zero level period, the velocity of the particles begins to decrease. At this time, the particles are no longer affected by torque but move with the flow of the fluid. Since the fluid is initially in a static state, the fluid slowly tends to become still at this time, and the velocity of the BNNSs also tends towards 0 m/s. However, at this time, the BNNSs still have a velocity, so the orientation angle continues to increase, and finally, a stepped waveform is formed.

**Figure 7:**
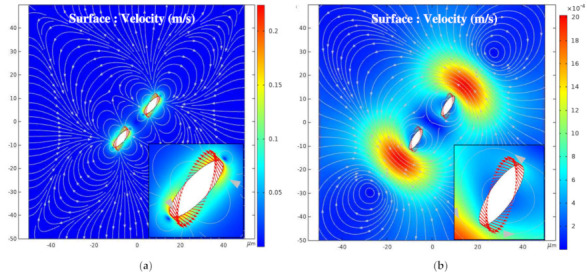
(**a**) Surface cloud map of fluid velocity during the high level; (**b**) surface cloud map of fluid velocity during the zero level.

Figure 7 shows a surface cloud map of the fluid velocity during the periods of high and zero pulse voltage when α = 45°. The streamline in the map represents the fluid flow velocity direction, and the arrow on the line represents the BNNSs velocity direction. Under the high level of the pulse voltage, the particles experience torque, and the velocity increases sharply to the maximum value, as shown by the blue curve in Figure 6. The movement of BNNS particles drives the flow of the ultrapure water fluid. At this time, the left and right sides of the fluid flow towards the middle, and the upper and lower sides flow towards the outside. Since the fluid is set to open boundary conditions in the simulation, the fluid can flow in and out freely. At the pulse voltage zero level, the particles no longer receive an external driving force, the flow rate slowly decreases, and the flow rate direction also changes accordingly. The fluid streamline becomes irregular, and the maximum velocity area spreads until it returns to a static state, as shown in Figure 7b. At this time, the BNNSs velocity is greatly reduced, but the movement direction has not changed. Therefore, the growth rate of the orientation angle gradually decreases, but the trend of increasing is not changed.

### 3.3. Influence of the Relative Angle β on the Global Arrangement

When BNNS particles move in an electric field, the presence of the particles will significantly change the local electric field and therefore change the mutual dielectrophoretic force between particles, which plays an important role in the global arrangement. Under different positions, the direction of the force generated by the dielectric interaction will change with time. The dielectric interaction of two particles under an electric field may be attractive or repulsive, depending on the relative angle β of the two particles.

In this section, the motion of two particles under different initial relative angles β is simulated. Set β = 0°, 15°, 45°, 75°, and 90°. Assuming that the particles have completed the local orientation process, set d = 2.0d* and α = 0°. From the previous analysis, the high level of the nanosecond pulse voltage can be regarded as a short-term DC voltage, so the dielectrophoretic motion of BNNSs in ultrapure water under the nanosecond pulse voltage is equivalent to the sequential action of many short-term DC voltages. The difference is the change in the velocity and direction of the movement during the zero level of the nanosecond pulse voltage. At this time, the movement of the particles depends on the flow of the fluid.

**Figure 8:**
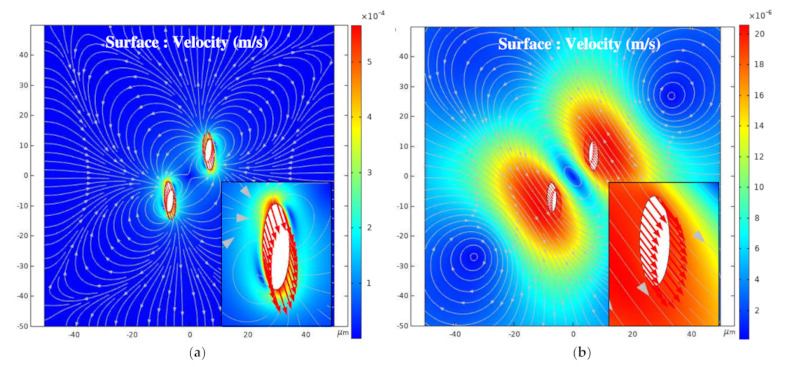
(**a**) Surface cloud map of the fluid velocity during the high level; (**b**) surface cloud map of the fluid velocity during the zero level.

**Figure 9:**
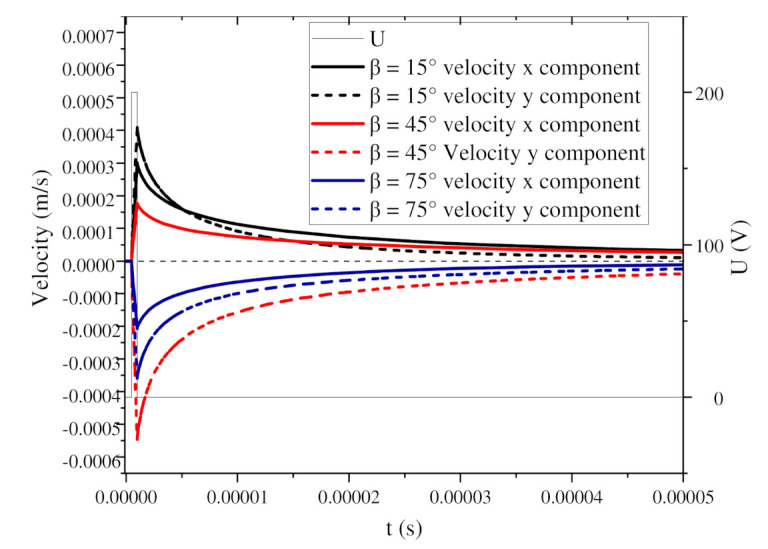
Change in velocity during 1 pulse voltage.

For example, when β = 45°, Figure 8a,b shows the surface cloud maps of the fluid velocity during the pulse voltage high level and zero level, respectively. The streamline in the map represents the fluid flow velocity direction, and the arrow on the line represents the BNNSs velocity direction. In Figure 8a, the BNNSs are subjected to a dielectrophoretic force, which generates a movement velocity, under the high level of the pulse voltage, so the movement velocity of the particles is relatively large, as shown by the red curve in Figure 9. The solid line represents the x component of the movement velocity when β = 45°, and the dashed line represents the y component of the movement velocity. According to the velocity change curve and Figure 8b, during the zero level, the flow rate slowly decreases, and the velocity that drives the BNNSs also slowly decreases; however, its velocity direction does not change during this period. Therefore, the distance between the two BNNSs still changes according to the changes during the high level, but the magnitude of the changes gradually decreases. In the case analyzed in this article, the zero level does not affect the overall movement trend of the BNNSs and is essentially no different from the movement when a DC voltage is applied.

Based on the previous theoretical analysis, the number of pulse voltages required to simulate global arrangement of particles is large, and the nanosecond pulse width causes the simulation step to be small, so simulating global arrangement of two particles under the pulse voltage takes a long time. To save simulation time, this paper simulates the movement of the particles under the first 100 pulse voltages and summarizes the rules. At the same time, the same-value DC voltage is used to obtain the changes in the trajectories and distance of the two BNNSs after the final global arrangement is reached.

#### 3.3.1. 0° < β < 90°

**Figure 10:**
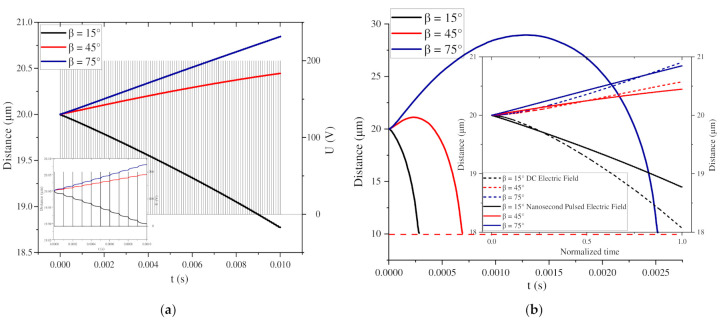
(**a**) Change in the distance at different β under 100 nanosecond pulse voltages; (**b**) change in the distance at different β under a DC voltage.

Due to the particularity of β = 0° and β = 90°, this section first discusses the other cases. Figure 10a,b shows the change in the distance between a pair of BNNS particles under 100 pulse voltages and a DC voltage at different β and d = 2.0d*.

Figure 10a shows the change in the distance between two particles of different β with time under the continuous action of the first 100 pulse voltages. When β = 45° and β = 75°, the distance between the two particles gradually increases, whereas the distance for β = 15° gradually decreases. The subfigure shows the change in the distance under the first 10 pulse voltages, showing a wave shape, indicating that the BNNSs continuously accelerate during the pulse voltage high level period and decelerate during the zero level period, but the velocity direction does not change.

**Figure 11:**
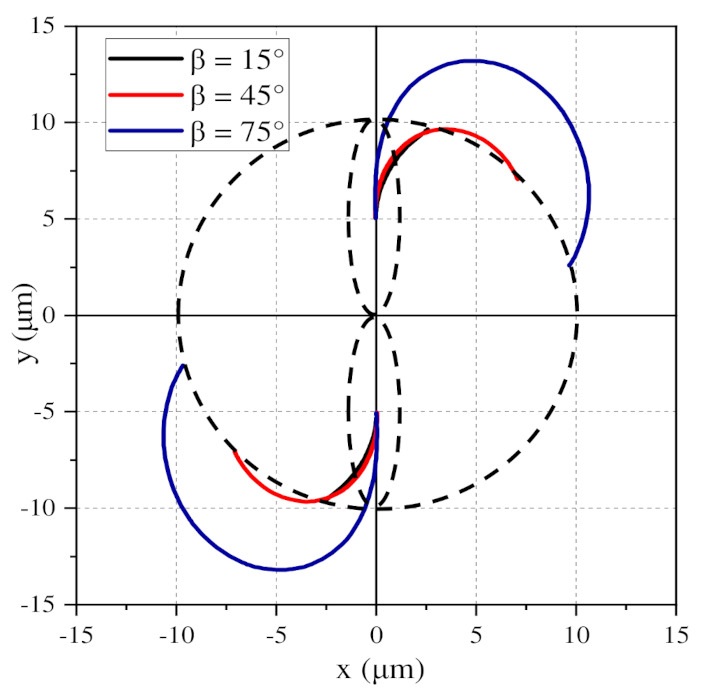
Motion trajectories of BNNSs at different β values under a DC voltage.

Figure 10b shows the change curve of the distance between two particles under the same-value DC voltage. Finally, the distance between the BNNSs becomes 10 µm, which completes the global arrangement into a chain, and the trajectories are shown in Figure 11. When β = 15°, the distance always decreases throughout the process, indicating that the two particles undergo attractive motion. When β = 45° and β = 75°, the distance first increases and then decreases. The increase for β = 75° is much larger than that for β = 45°. The results show that when β = 75°, the repulsion of the two particles is stronger than that for β = 45°, and when β = 45°, the movement becomes an attractive motion in a shorter time.

The subfigure in Figure 10a shows the comparison of the distance changes between the nanosecond pulse voltage and DC voltage when moving to the same position. The solid line represents the change in the distance between the particles under the nanosecond pulse voltage, and the dashed line represents the change in the distance at the initial time under the DC voltage. The times of the two curves are normalized, and the change trends of the two curves are the same. This shows that the distance change under 100 pulse voltages is similar to that at the initial time under a DC voltage, except that the time required for the continuous DC voltage is greatly shortened. At the same time, it also indirectly shows that the zero level of the pulse voltage does not change the direction of the particle movement velocity, which is consistent with the previous analysis. Therefore, the change trend of the particle distance under the DC voltage can be used to indirectly indicate the trend under the pulsed electric field, that is, the chain structure is finally formed.

**Figure 12:**
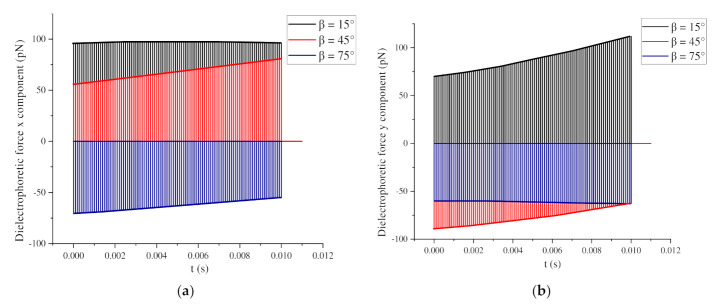
(**a**) Changes in the x component of the BNNSs dielectrophoretic force; (**b**) changes in the y component of the BNNSs dielectrophoretic force.

The movement of BNNS particles under a pulse voltage depends on the force conditions, as shown in Figure 12a,b, which displays the changes in the dielectrophoretic force under 100 pulsed electric fields when β = 15°, 45°, and 75°. When β = 15°, the x and y components of the dielectrophoretic force on the particles are both positive, the y component of the dielectrophoretic force gradually increases, and the x component is basically unchanged, indicating that an attractive force exists between the two particles. With the action of the nanosecond pulsed electric field, the attractive force gradually increases, so the distance between the two particles gradually decreases.

When β = 45°, the x component of the dielectrophoretic force on the particles is positive, but the y component is negative. With the action of the nanosecond pulsed electric field, the dielectrophoretic force in the x direction gradually increases, while that in the y direction gradually decreases. At this time, the two particles are repelled, so the distance gradually increases. However, when the dielectrophoretic force in the y direction becomes positive, the two particles attract each other, and the distance gradually decreases. When β = 75°, the x and y components of the particle dielectrophoretic force are both negative. At this time, a repulsive force exists between the particles, and compared with β = 45°, the repulsive force is greater; thus, the increase in the distance is greater.

#### 3.3.2. β = 0° and β = 90°

**Figure 13:**
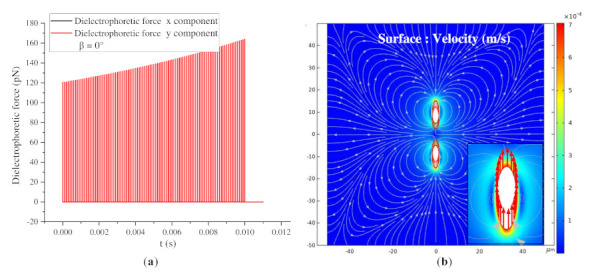
(**a**) Dielectrophoretic force change of BNNSs under pulse voltages (β = 0°); (**b**) surface velocity cloud map after 100 pulse voltages (β = 0°).

**Figure 14:**
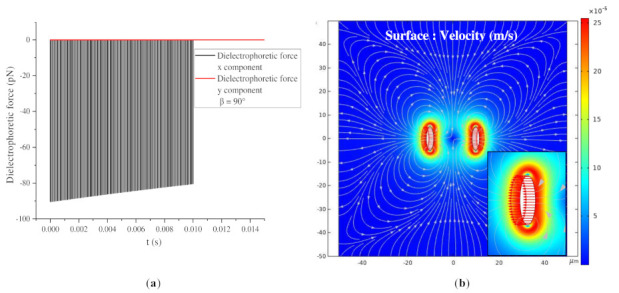
(**a**) Dielectrophoretic force change of BNNSs under pulse voltages (β = 90°); (**b**) surface velocity cloud map after 100 pulse voltages (β = 90°).

According to the analysis in Section 3.3.1, the BNNSs are subjected to dielectrophoretic forces in the x direction and y direction when β = 15°, 45°, and 75°. However, two special cases arise. When β = 0°, the long axis of the particle is parallel to the electric field direction. During the movement, a dielectrophoretic force only exists in the y-direction, and the dielectrophoretic force in the x direction is almost 0. An attractive force exists between the two particles, as shown in Figure 13. When β = 90°, a dielectrophoretic force only exists in the x-direction, and the dielectrophoretic force in the y-direction is almost 0. A repulsive force exists between the two particles. With the application of the pulse voltage, the two particles move away from each other, and the dielectrophoretic force gradually decreases, as shown in Figure 14.

Figure 13b shows a surface velocity cloud map after 100 pulse voltages corresponding to (a), and the direction of the arrow indicates the particle velocity direction. When β = 0°, the direction along which the BNNSs receive the dielectrophoretic force is parallel to the electric field direction, so their velocity direction is also parallel to the electric field. The subgraph indicates that the movement velocity of the lower BNNSs in the map is upward, while the velocity of the upper BNNSs is downward. Therefore, the two particles are attracted to each other, become close to each other, and drive the flow of the ultrapure water fluid. The flow field and pressure around the two particles change with their positions. The fluid streamlines are symmetrical, with the left and right ends flowing outward and the upper and lower ends flowing inward.

When β = 90°, the direction of the dielectrophoretic force on the BNNSs is perpendicular to the electric field, so their velocity direction is also perpendicular to the electric field. The subgraph indicates that the velocity of the BNNSs on the left side of the figure is to the left, while the velocity of the BNNSs on the right is to the right. Therefore, the two particles repel each other and move away from each other, driving the flow of the ultrapure water fluid, and the flow field and pressure around the two particles change with their positions. The fluid streamlines are symmetrical, with the left and right ends flowing outward and the upper and lower ends flowing inward. At this time, the fluid flow direction is almost the same as when β = 0°. As the distance between the two particles increases, the dielectrophoretic interaction force between the particles becomes weaker.

## 4. Conclusions

This research is the first to simulate and analyze the dielectrophoretic motion of BNNS particles in ultrapure water under a nanosecond pulse voltage and propose a simulation theory of particle dielectrophoretic motion under a pulse voltage. The results show that when the pulse voltage is applied, the relaxation polarization time of the particles and the fluid needs to be considered and compared with the nanosecond pulse width. This is the fundamental difference from a direct voltage. In this paper, the relaxation polarization time of BNNS particles and ultrapure water is much shorter than the nanosecond pulse width, so the high level of the pulse voltage can be regarded as a short DC voltage.

On this basis, the influence of different self-angles α on the local orientation and different relative angles β on the global arrangement of two BNNS particles is considered, and the dielectrophoretic motion law under a pulse voltage is deeply analyzed. The results show that the particles are oriented or moved by the dielectrophoretic force during the high level of the pulse voltage, which changes the orientation angle or distance. During the zero level period, the particles experience no dielectrophoretic force and follow the natural movement of the fluid without the particle movement direction changing. Therefore, the orientation angle and distance changes show step-like and wave-like curves, respectively.

The theory of particle dielectrophoretic motion under a pulse voltage proposed in this paper can provide a theoretical basis for experiments on pulsed electric field-induced particle orientation and arrangement. The simulation model can be used as an effective tool for the simulation of any dielectric whose relaxation polarization time is much shorter than the pulse voltage width. To clarify the influence of different positions on the dielectrophoretic motion of particles under a pulse voltage, in the next work, we will continue to explore the simulation with epoxy resin as the fluid. At this time, the preconditions of the simulation will not be met, and further analysis will be needed to establish a more complete theory and obtain a more comprehensive law of motion.

## Data Availability

The data presented in this study are available on request from the corresponding author.
